# Uveitis Sarcoidosis Presumably Initiated After Administration of Shingrix Vaccine

**DOI:** 10.7759/cureus.4920

**Published:** 2019-06-17

**Authors:** Milad Heydari-Kamjani, Ivanka Vante, Paven Uppal, Michelle Demory Beckler, Marc M Kesselman

**Affiliations:** 1 Osteopathic Medicine, Nova Southeastern University Dr. Kiran C. Patel College of Osteopathic Medicine, Fort Lauderdale, USA; 2 Family Medicine, Nova Southeastern University, Dr. Kiran C. Patel College of Osteopathic Medicine, Fort Lauderdale, USA; 3 Miscellaneous, Nova Southeastern University, Dr. Kiran C. Patel College of Osteopathic Medicine, Fort Lauderdale, USA; 4 Immunology, Nova Southeastern University Dr. Kiran C. Patel College of Osteopathic Medicine, Fort Lauderdale, USA; 5 Rheumatology, Nova Southeastern University Dr. Kiran C. Patel College of Osteopathic Medicine, Fort Lauderdale, USA

**Keywords:** shingrix, sarcoidosis, uveitis, autoimmune, as01b adjuvant

## Abstract

Uveitis is a form of intraocular inflammation that can occur as a result of infection, injury, vaccination, or as a systemic manifestation of autoimmune or inflammatory diseases including sarcoidosis. Sarcoidosis is an inflammatory disease that involves the formation of abnormal granulomas in multiple organ systems. The hallmark of sarcoidosis is a non-caseating granuloma seen on biopsy. Here, we present a case report of a healthy 56-year-old Caucasian female who presented with uveitis sarcoidosis that was presumably initiated after administration of the Shingrix vaccine. Shingrix is a new attenuated subunit vaccine for Varicella Zoster Virus with an AS01B adjuvant that can result in a potent immune response. The Shingrix vaccine is made using Chinese hamster ovary cells which can contaminate the final vaccination product. Together, through the process of molecular mimicry and strong induction of the immune response, administration of Shingrix may have caused or exasperated this patient’s autoimmune etiology.

## Introduction

Uveitis is a form of intraocular inflammation involving the uveal tissues (middle layer of the eyewall) including the iris, ciliary body, and choroid [1]. Uveitis can occur as a result of infection, injury, vaccination, or as a systemic manifestation of autoimmune or inflammatory diseases including sarcoidosis [1]. Sarcoidosis is an inflammatory disease that involves the formation of abnormal masses or nodules (granulomas) consisting of inflamed tissues, affecting multiple organ systems throughout the body [2]. The presence of uveitis among patients with sarcoidosis ranges between 25% and 50% [3]. Patients with uveitis commonly present with blurred vision, floaters, eye pain, redness, abnormal sensitivity to light, and headaches [1]. Diagnosis is made by physical examination, funduscopic exam, ocular pressure test, and/or a slit lamp exam [1]. The hallmark of sarcoidosis is non-caseating granuloma seen on biopsy [4]. Presumably, exposure to some unknown environmental antigen triggers an exaggerated cellular immune response in target organs, promoting the development and accumulation of granulomas [5]. Independent of the cause of uveitis, treatment commonly involves the use of steroids, either eye drops or injections, but other options including immunosuppressive agents and surgery may be used dependent on the degree and type of inflammation [1]. Here, we present a case report of a healthy 56-year-old Caucasian female who presented with uveitis sarcoidosis that was presumably initiated after the administration of the Shingrix vaccine.

## Case presentation

A 53-year-old Caucasian, female patient was referred to Rheumatology after she presented to her primary care physician with complaints of headaches, left eye redness, photophobia, and eye pain. The patient was referred to Ophthalmology. She was managed with ophthalmic corticosteroids that resulted in the resolution of her symptoms, but this ophthalmology consult was non-diagnostic as to etiology. Subsequently, her symptoms recurred a second time in the right eye, and this time, the Ophthalmology evaluation revealed granulomatous uveitis. The patient was treated with prednisone acetate eye drops which resulted in resolution of her uveitis.

The patient’s past medical history is significant only for mild persistent asthma managed with inhaled Beta-2-agonist. The patient reported a history of knee and ankle swelling which she attributed to walking and running. Physical examination did not reveal any joint, skin, pulmonary or cardiac findings to suggest an underlying connective tissue etiology. She denied recent fever, rash, or tick bite. Interestingly, she reported receiving the Shingrix vaccine four days prior to her initial eye complaints.

Laboratory results showed elevated levels of 1,25-OH-Vitamin-D levels (83 pg/ml), angiotensinogen converting enzyme (ACE) (86U/L), and calcium ions (10.7 mg/dl) and a positive rheumatoid factor (RF). Repeat ACE level after resolution of uveitis was normal. Initial radiographic series suggested chronic obstructive pulmonary disease. Subsequent high-resolution computed tomography (HRCT) identified the presence of bilateral hilar and mediastinal adenopathy. Overall, the presence of uveitis, elevated laboratory values, and bilateral hilar and mediastinal adenopathy on HRCT are suggestive of dormant granulomatous disease, presumably sarcoidosis that was possibly set off by the administration of the Shingrix vaccine.

She is currently being treated with ophthalmic corticosteroid medication. Systemic corticosteroid was not indicated since she is asymptomatic from a rheumatological point of view. She also received the second dose of Shingrix vaccine with no further reoccurrence of uveitis. The suppression of uveitis can be explained by her current ophthalmic corticosteroid medication. The patient will continue to be tapered off ophthalmic corticosteroids. If there is a reoccurrence of uveitis, a biologic therapy, such as adalimumab may be considered.

## Discussion

Currently, the international criteria for the diagnosis of ocular sarcoidosis (IWOS) includes “four levels of certainty” [6]. These are defined as: Definite, Presumed, Probable or Possible based on seven intraocular signs and five laboratory investigations. If a patient with uveitis suggestive of sarcoidosis is also found to have bilateral hilar adenopathy on chest imaging, but without a confirmatory biopsy, the patient can be presumed to have ocular sarcoidosis without a definitive diagnosis [6]. Based on the IWOS criteria, our patient is considered a “presumed” diagnosis of ocular sarcoidosis. Interestingly, our patient experienced two episodes of uveitis four days after being administered the Shingrix vaccine.

Shingrix is a new attenuated subunit vaccine for Varicella Zoster Virus (VZV) containing the VZV glycoprotein-E formulated with AS01B adjuvant [7]. The efficacy of this vaccine was demonstrated in clinical trials in adults ≥ 50 years (ZOE-50) and adults ≥70 years (ZOE-70). These studies showed that Shingrix is remarkable efficacious by about 97% in preventing shingles. This vaccine has also been studied in the immunocomprised population, such as those with HIV or cancer or transplant patients. However, patients with autoimmune etiologies such as rheumatoid arthritis, psoriatic arthritis, ankylosing spondylitis, and lupus were excluded in the ZOE-50 and ZOE-70 clinical trials.

There are two main reasons why Shingrix might be contraindicated for autoimmune disease patients. First, this is the first time this type of adjuvant is being used in human vaccination and it contains a combination of two ingredients, in particular, saponin derived QS21 molecule and 3-O-desacyl-40 -monophosphoryl lipid A (MPL) [7]. QS21 is a molecule extracted from the South American tree. MPL is a lipopolysaccharide derived from the Salmonella minnesota. Together, this combination can result in a potent stimulation of both classical and monocyte-derived dendritic cells and, thereby, it may enhance antigen presentation to T cells [7]. Moreover, multiple in vivo studies have shown that MPL derived from Salmonella minnesota can induce uveitis [8], suggesting a direct role of AS01B adjuvant in promoting inflammatory reactions. Given the high level of immune reactivity seen in autoimmune disease patients, this adjuvant may contribute to increased activity of adaptive immune responses in this cohort of patients. Second, it is important to note that the Shingrix vaccine is made using Chinese hamster ovary (CHO) cells [7]. Therefore, despite high purification standards, the vaccine likely has a small amount of host cell proteins (HCP) in the final product. If the homology between HCPs and self-peptides are less than 100%, these peptides may be expressed on low-affinity self-reactive (LASR) T cells and be positively selected in the thymus, leading to the proliferation of T cells with a high affinity to self-peptides [9]. Consequently, the presence of contaminated animal proteins in vaccines can stimulate autoimmunity due to molecular mimicry.

Lastly, in a review of the literature, a prior study indicated that keratitis with a secondary bacterial infection was one of the serious adverse effects in study participants that received at least one dose of the Shingrix vaccination [10].

Collectively, analysis of Shingrix clinical trials and its proprietary adjuvant warrants questioning of its safety in patients with a heightened immune response. Specifically, physicians involved in the care of patients with autoimmune disease have expressed a concern that the administration of Shingrix vaccine may trigger or exacerbate autoimmune etiologies. As a result, at this point, we cannot exclude the possibility of the Shingrix vaccine as a trigger of uveitis-induced sarcoidosis in this patient.

## Conclusions

Patient declined hilar biopsy which limited a definitive diagnosis of sarcoidosis. Shingrix is a new attenuated subunit vaccine for Varicella Zoster Virus with an AS01B adjuvant that is used for the first time in human vaccination. The Shingrix vaccine is made using Chinese hamster ovary (CHO) cells which can contaminate the final vaccination product. The AS01B is a proprietary adjuvant that can result in a potent immune response. Together, through the process of molecular mimicry and strong induction of the immune response, Shingrix may cause or exasperate autoimmune etiology. As a result, at this point, we cannot exclude the likelihood of the Shingrix vaccine as a trigger of uveitis-induced sarcoidosis in this patient.
